# Hepatitis B Birth Dose Vaccination among Vietnamese Children: Implications for the Expanded Program on Immunization

**DOI:** 10.1155/2019/3453105

**Published:** 2019-06-16

**Authors:** Hao Nguyen Si Anh, Hoang-Long Vo, Long Hoang Bao, Hien Tran Minh, Ha Tran Thi Thu, Vu Duy Kien

**Affiliations:** ^1^Institute for Preventive Medicine and Public Health, Hanoi Medical University, Hanoi, Vietnam; ^2^Institute of Gastroenterology and Hepatology, Hanoi, Vietnam; ^3^Oncare Medical Technology Company Limited, Hanoi, Vietnam

## Abstract

**Background:**

This study assesses the prevalence of Vietnamese children receiving the hepatitis B (HepB) vaccine birth dose and explores its associated socioeconomic factors.

**Methods:**

We used the data of the Multiple Indicator Cluster Survey, 2014. We estimated the overall percentage of HepB birth dose vaccination among 0–23-month-old children and its percentages according to selected characteristics. Multiple logistic regression was applied.

**Results:**

62.8% of children received the HepB vaccine birth dose. The prevalence rates by selected factors ranged from 35.3% to 76.7%. The categories with the lowest prevalence rates were children who had low birth weight (41.6%), had a mother aged less than 20 years (35.3%), had a mother with primary or less education (42.7%), belonged to ethnic minorities (30.3%), resided in rural areas (59.9%), and were in the 1^st^ quintile of mother's socioeconomic status (38.6%). Receiving HepB vaccine birth dose was associated with child's birth weight, mother's age, mother's education, socioeconomic status, and ethnicity.

**Conclusions:**

This study identified vulnerable groups, upon which policy-makers should focus their efforts to equitably and sustainably tackle birth dose HepB vaccine coverage as well as the full vaccination coverage, thereby promoting long-lasting herd immunity in this country.

## 1. Introduction

Hepatitis B (HepB) is an infection of the liver caused by the HepB virus that attacks liver cells [1]. The HepB virus is transmitted by exposure to blood or other bodily fluid of an infected person. HepB results in more than 780,000 deaths every year, mostly from liver cancer and cirrhosis, while an estimated 257 million people are living with chronic HepB [2]. HepB is considered as a major global health problem, especially in the developing countries such as Vietnam [3, 4].

The Western Pacific region is well-known as an area with a very high burden of HepB, accounting for nearly half of all chronic HepB cases worldwide [3, 4]. Vietnam, with about 8.6 million people who are carriers of virus, is one of 11 countries in the Western Pacific region with the highest prevalence of HepB [5, 6]. Previous studies in different regions in Vietnam showed that the incidence of HepB has been estimated approximately 10%-20% of the population, in which the viruses are mainly vertically transmitted from mother to child [7–9]. Most people currently living with HepB virus infection were born before HepB vaccine was widely available and used in the infancy [2].

The HepB birth dose is a monovalent vaccine containing surface proteins of the HepB virus absorbed by aluminum hydroxide or monophosphoryl lipid A adjuvant [10]. Surface antigens extracted from HBsAg-positive human plasma are asymptomatic or recombinant in yeast cells [10, 11]. In 2006, following the World Health Organization (WHO) recommendation, Vietnam officially implemented the first dose of HepB vaccination for the infants within the first 24 hours after birth [12]. A study conducted in 51 provinces across Vietnam revealed that the rate of positive children with HBsAg in the period of 2008-2011 fell to less than 2% compared to 3.62% during 2000-2003 [5, 13]. The coverage of HepB vaccination within first 24 hours after birth also reached 76.6% at the end of 2017 after 3 decades of the Vietnamese government's efforts [6].

In Vietnam, the Kinh is the main ethnic group, and other ethnic groups account for up to 14% of the national population [14]. While Vietnam is putting more and more effort to maintain the coverage of HepB birth dose, the country continues to face the problem of “vaccine hesitancy”, one of the global health threats [15]. More than 8.8% of Vietnamese women and 12.3% of Vietnamese men carry viruses for coming decades [1, 5]. These are considered as great challenges that require enduring and comprehensive efforts for Vietnam to achieve the goal of “HBsAg prevalence of less than 0.1% in 5-year-old children” for the period 2018-2030 control [6].

Recognizing the neglected aspects of the HepB birth dose coverage across the country may contribute to developing policy to expand the prevention interventions for mother-to-child transmission of HepB virus. Exploring key socioeconomic factors that are as the barriers to the HepB birth dose vaccination could pave pathway for scaling up other specific healthcare interventions and therefore could contribute to investigation, prevention, and management of healthcare-associated HepB infections among both mothers and newborns in the community. In the present paper, we used the most updated data from the Multiple Indicators Cluster Surveys (MICS) of the United Nations International Children's Emergency Fund (UNICEF) in the survey round of 2014 to assess the prevalence of Vietnamese children who received the birth dose of HepB vaccination and to identify socioeconomic factors associated with receipt of the birth dose.

## 2. Methods

### 2.1. Data Source

Data from the MICS 2014 was used for this study. The MICS was conducted by the General Statistics Office in collaboration with the Ministry of Health and the Ministry of Labor, Invalids and Social Affairs. Financial and technical supports for the survey were provided by the United Nations Children's Fund and the United Nations Population Fund. The MICS is nationally representative survey covering a broad range of issues affecting the health, development, and living conditions of Vietnamese women and children. The number of children aged 0-23 months included in the 2014 MICS was 1382 [16].

### 2.2. Variables and Indicators

The main outcome variable in our study was a binary variable specifying whether a 0–23-month-old child received the first dose of HepB vaccination within 24 hours after birth. Data for the available were extracted retrospectively from the MICS 2014. In these surveys, data regarding the HepB birth dose vaccination were obtained from vaccination cards. If no vaccination card was available, the interviewers would ask mothers whether their child received a vaccination against HepB first dose vaccination [12, 16].

The explanatory variables are as follows: child's sex (male/female), low birth weight defined as less than 2,500 grams (yes/no), mother's age (<20/20-35/36-49), mother's education (primary or less/lower secondary/upper secondary and higher), ethnic group (Kinh/Hoa ethnicity and minority ethnic group), living area (rural/urban), and mother's socioeconomic status.

Mother's socioeconomic status (known as household's socioeconomic status) was measured as an asset-based wealth index and was constructed using principal component analysis (PCA). The MICS dataset included the Household Wealth Index which was calculated by the GSO of Vietnam. The index was based on the ownership of consumer goods, dwelling characteristics, water and sanitation, and other characteristics related to household wealth. Weights (factor scores) were assigned to correspond with individual household assets [16]. The details of the method used for estimating the wealth asset index are described elsewhere [16]. Five categories (quintiles) were ranged from the poorest to the richest.

### 2.3. Data Analysis

Both descriptive and analytical methods were used in the present paper. We estimated the overall percentage of HepB birth dose vaccination among 0–23-month-old children and the percentage according to sex, region, area, ethnicity, mother's education, and socioeconomic status. The Wald's Chi-square test was applied to compare the differences of receiving the HepB dose vaccination within 24 hours after birth among groups. The multiple logistic regression was used for the binary primary outcome variable to explore the factors associated with HepB birth dose vaccination. The cumulative probability of being vaccinated among ethnicities at age t among ethnic groups was estimated by the inverse Kaplan–Meier survival function (or 1−SKM(t)), also known to measure the fraction of children receiving the birth dose HepB vaccination for a certain amount of time [17]. All statistical analyses were carried out using Stata® 13.1 (StataCorp LLC, USA), with weighting factors for children from the dataset. A significance level of p-value <0.05 was used.

### 2.4. Research Ethics

This study was conducted on secondary data from the MICS with all identifiable information removed. The survey had obtained informed consent from the mothers before administering survey questionnaires and the consenting process had stated that data can be analyzed in subsequent analyses without retaking informed consent. All information in the original dataset was collected confidentially.

## 3. Results

### 3.1. Prevalence of Receiving the Hepatitis B Birth Dose Vaccination

Prevalence rates and 95% confidence intervals for receiving the birth dose of HepB vaccination in each category by the selected socioeconomic factors are presented in Table 1. In 2014, 62.8% of children received the birth dose of HepB vaccination. The prevalence rates by selected factors ranged from 35.3% to 76.7%. The categories with the lowest prevalence rates were children having low birth weight (41.6%), had a mother aged less than 20 years (35.3%), had a mother with primary or less education (42.7%), belonged to ethnic minorities (30.3%), resided in rural areas (59.9%), and were in the 1^st^ quintile mother's wealth status (38.6%). The difference in prevalence rates among all categories of each factor were significant (p-value <0.05) for most selected socioeconomic factors (except for the children gender).

**Figure 1:**
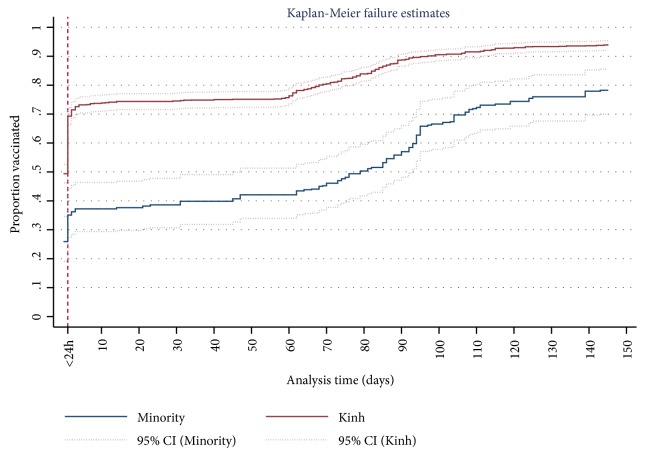
Cumulative proportions of receiving the birth dose of HepB vaccination by age in days among Vietnamese children belonging to Kinh/Hoa ethnicity and minority ethnic groups.

The two curves of cumulative proportion for children receiving the birth dose of HepB vaccination belonging to Kinh/Hoa ethnicity and belonging to minority by children age in days are shown in Figure 1. For Kinh/Hoa children, on birth day the birth dose vaccination rate started at 69.3%, then the birth dose rate rose sharply to above 90% at age 120 days and the following days (red curve in Figure 1). For minority, at age 1 day, more than 35% children received the birth dose, then the proportion of receiving the birth dose increased to 80% at age 145 days and following days (blue curve in Figure 1). However, the wide gap of receiving HepB vaccination between Kinh/Hoa and minority was still significant (Figure 1).

### 3.2. Socioeconomic Factors Associated with HepB Birth Dose Vaccination

Compared with the prevalence of receiving the birth dose of HepB vaccination among children with low birth weight, the odds was twice higher for the children with normal birth weight (OR 2.13; 95%CI 1.16-3.89). Kinh/Hoa children had significantly higher odds of HepB birth dose vaccination than individuals from ethnic minorities (OR 3.15, 95%CI: 2.04–4.88). Mother's age was significantly associated with increased prevalence of receiving the HepB birth dose vaccination for their children. Children whose mothers had higher education were significantly more likely to have had completion of the HepB birth dose vaccination compared with those had mothers experiencing primary or less education. The odds of HepB birth dose vaccination were higher in children who belonged to the families with better economic status (Table 2).

## 4. Discussion

In a Kate Whitford et al.'s report (2018), most immunization schedule in all studies included a first dose of HepB vaccine within 24 hours after birth except for one from Italy [18], where the birth dose was only included in the targeted vaccination schedule [19]. In Vietnam, in the decision number 2620/QD-BYT guiding the implementation of birth dose HepB vaccination in 2012, the Ministry of Health set the goal that the immunization coverage of HepB birth dose should reach at least 65% [20]. In our study, the prevalence of receiving HepB vaccine birth dose within 24 hours (62.8%) did not reach the target. However, it reported a consistently increasing trend since the HepB vaccine dose was recommended to be given within 24 hours after birth in 2006. The WHO has reported that the global HepB immunization coverage with three doses during infancy reached 84% in 2015 [21]; nevertheless, the global birth dose coverage of HepB vaccine remained low, at an estimated 39% in 2015 [22]. So, the present proportion of timely HepB birth dose vaccination among Vietnamese children was overall acceptable. This finding was important for the Vietnam's national immunization program to recognize the existing gap which might result in the vaccination proportion not reaching the goal set by the Ministry of Health, thereby continuously improving the immunization service in highly endemic countries like Vietnam, where 10.5% of pregnant women are HepB virus carriers [23] as well as where a high proportion of HepB virus infections are acquired perinatally [24, 25].

In our study, low birth weight children had significantly lower vaccination rates for HepB within 24 hours after birth. Saari et al. (2003) reported that vaccination rates could be affected by the infant's birth weight [26]. Infants with a birth weight of below 2500 grams may have weaker immune response; therefore, the HepB vaccine first dose cannot be requested by their doctors [27].

In our study, the prevalence of receiving HepB vaccine dose within 24 hours after birth was significantly higher in urban areas than in rural areas. Previous studies in Vietnam and elsewhere also found that urban areas had higher coverage of full immunization [28, 29]. This may be explained by higher availability and better quality of vaccination services in urban areas through concentrated efforts by the organizations and individuals dealing with vaccination service. Other explanations may be difficult logistically, particularly for home deliveries as cold-chain infrastructure is limited in remote rural areas. There are two types of vaccinations known as expanded vaccination (free-of-charge) and service vaccination (parents have to pay for vaccination) in Vietnam. Public health facilities having their assigned functions and tasks of vaccination for all children in the expanded immunization program (EPI) are compulsorily available throughout this country. Nevertheless, private immunization service centers, which are also allowed to register with the local Department of Health to implement vaccination in the EPI, are opened more in urban areas where the citizens have better socioeconomic status. To our knowledge, in rural areas known to have lower socioeconomic status, the uptake of HepB birth dose vaccination at most commune health centers and general hospitals may be due to the free-of-charge vaccination.

In Vietnam, there is a fact that women under the age of 20, who are in attending-school age, are not able to get married and give birth later. On the other hand, a finding of Linh Cu Le et al. reported that a risk of unintended pregnancy was 1.5 times higher in Vietnamese women marrying before 20 than those later or early marriage associated with unintended pregnancy [30]. Therefore, their awareness of raising a child can be incomplete. Perhaps the reasons above are appropriate to explain one of the present findings that children having mothers less than 20 years old were less likely to receive HepB vaccine birth dose compared with those having mothers aged over 20 years. The gap between the rich and the poor, between Kinh and other ethnic minority groups, between low educational level and high one, and between ages is concerning issues in Vietnam [14, 31]. Also in this study, we found that mother's education, household wealth, and mother's ethnicity were associated with timely immunization completion for HepB vaccine birth dose in the multivariate analysis. In particular, children of Kinh/Hoa ethnicity were more likely to receive timely immunization completion for HepB vaccine birth dose compared with children from minority ethnic groups. This difference might be due to the fact that people of different ethnicity had different attitudes, health-seeking behaviors, and socioeconomic status. In Vietnam, the Kinh who live mainly in the plains, near the rivers, and in urban areas are more likely to benefit from better socioeconomic conditions [32]. On the other hand, most ethnic minority groups, who generally have relatively poorer socioeconomic conditions and lower literacy rate, are living in the highlands and rural and mountainous areas [32]. Hence, women from ethnic minority groups have difficulties in approaching in expanded immunization services like the HepB birth dose vaccination. We found that although the proportion of receiving HepB vaccine birth dose for both Kinh/Hoa ethnicity and ethnic minority groups gradually increased at following days, these figures for timely vaccination within 24 hours as recommended were low and in particular for HepB birth dose vaccination rate of ethnic minority groups were very low, at estimated below 40%. According to issued document in the 9th National Congress, equity among all ethnic groups was recognized in The Vietnamese Constitution as a priority [14]. This finding is important for this country's government as well as policy-marker to understand the barriers to preventing mothers' access to HepB birth dose vaccination. The present finding suggests that narrowing the gap between the Kinh/Hoa ethnicity and minority ethnic group is still a long-lasting way in the development and implementation of a strategy to improve timely birth dose coverage for HepB vaccine, in particular for a low-middle-income country like Vietnam. The poverty gap among ethnic groups has been documented in a previous report by Hai-Anh Dang, suggesting that the vaccination rates for Vietnamese children need to increase among ethnic minority women, considered as the short-term approach [33]. Therefore, our finding provides important evidence for the scientists and policy-makers in advancing the accessibility for HepB vaccine first dose within 24 hours after birth, which contributes to the full immunization coverage in the EPI.

The findings from this study have meaningful policy implications. This is the first study that assessed the coverage of timely HepB vaccine birth dose according to socioeconomic factors using the MICS data, which are the national representative immunization survey data in Vietnam. Importantly, the resource mobilization for the immunization in Vietnam is currently limited, not meeting the demand of expanded immunization [34]. In the context of Vietnam being a middle-income country, international funding support for expanded immunization is declining [34]. Hence, Vietnam has faced the challenge to meet the huge demand for the investment resources for expanded vaccination, including the consolidation and supplementation of cold-chain equipment for the vaccine preservation and the training of qualified health workers in the immunization [34]. This study identified vulnerable population groups (children with low birth weight, living in rural areas, mothers aged less than 20 years, mothers with low education, mothers from ethnic minorities, and poor socioeconomic status), upon which the policy-makers should focus their efforts to equitably and sustainably tackle the inequalities in the receipt of HepB birth dose as well as the full vaccination coverage, thereby promoting long-lasting herd immunity in Vietnam. Also based on the important findings in the present study, the public health decision-makers can understand that identified vulnerable populations above are underserved, which helps them consider integrating vulnerable populations-related issues of vaccination into existing national programmes for newborn HepB vaccination at four administrative levels of health establishments (central level, provincial level, district level, and commune), as well as building the evidence-based guidance of priority actions for the vaccination. Country policy-makers and immunization program implementers should consider the fact that the limited resources towards the Vietnam EPI need allocating most effectively in remote mountainous areas where the main habitats of disadvantaged populations exist.

However, we acknowledge some limitations to this study. First, due to the limitation of the cross-sectional study design of the MICS, the results should be interpreted with caution so that they are not interpreted as implying causality. Second, the estimations of receiving HepB vaccine birth dose were derived from available information on vaccination cards and/or reported by mothers. Therefore, some children may have been vaccinated but did not have immunization card and were excluded from the study. In addition, mothers may have forgotten to report the vaccination of their children during their interviews. These would underestimate the birth dose of HepB vaccination coverage in this study. Finally, the cultural aspects related to uptake of HepB birth dose vaccination could not be assessed, such as acceptability and attitudes of Vietnamese women towards the vaccine quality.

## 5. Conclusions

We found that the prevalence of receiving the first dose of HepB vaccine within 24 hours after birth did not meet the target of Vietnam (the immunization coverage of HepB birth dose should reach at least 65%). It is important to continue to coordinate with the WHO to support the implementation of newborn HepB vaccination; nevertheless, the targeted interventions in vulnerable population groups including child's low birth weight, mother's age less than 20, mother's low education, mother's low socioeconomic status, and child's ethnicity should be prioritized. There was a significant gap in the HepB vaccine birth dose coverage between the Kinh/Hoa ethnicity and minority ethnic groups, suggesting a need to improve both access and demand for HepB vaccine after birth among the other ethnic groups, in particular for minority ethnic groups living in remote and poor conditions. Furthermore, the policy developments in the HepB birth dose vaccination in particular and recommended vaccinations of the EPI in general should be established based on vulnerable populations, which leads to the sustainable interventions to decrease risk for healthcare-associated infections.

## Figures and Tables

**Table 1:** Prevalence of receiving timely the birth dose of hepatitis B vaccination by selected socioeconomic factors among Vietnamese children aged 0–23 months, MICS 2014 (n=1382).

Characteristics	Un-weighted sample size	Weighted prevalence	Chi-Square
	n (weighted %)	% (95%CI)	p-value
Sex			0.7
Male	741(54)	62.3(57.6,66.7)	
Female	641(46)	63.4(58.8,67.7)	
Low birth weight			<0.01
No	1311(95.4)	63.8(60.2,67.3)	
Yes	71(4.6)	41.6(29.2,55.2)	
Mother's age			<0.01
<20	91(5.5)	35.3(25.0,47.3)	
20-35	1131(83.4)	64.6(60.8,68.2)	
36-49	160(11.1)	62.7(53.8,70.8)	
Mother's education			<0.01
Primary or less	259(16.1)	42.7(34.0,51.8)	
Lower secondary	479(36.4)	63.6(58.6,68.4)	
Upper secondary and tertiary	644(47.5)	68.9(64.5,73.1)	
Ethnicity			<0.01
Kinh	1062(83.7)	69.1(65.6,72.4)	
Minority	320(16.3)	30.3(23.7,37.9)	
Area			<0.01
Urban	526(29.8)	69.6(64.5,74.3)	
Rural	856(70.2)	59.9(55.2,64.4)	
Mother's wealth status			<0.01
1st quintile (poorest)	329(19.3)	38.6(31.3,46.4)	
2nd quintile	245(19.6)	66.2(59.2,72.6)	
3rd quintile	250(20.1)	62.2(55.5,68.5)	
4th quintile	284(21.5)	76.7(69.8,82.4)	
5th quintile (richest)	274(19.5)	68.5(62.1,74.3)	

Overall	1382(100)	62.8(59.2,66.3)	

CI: confidence interval.

**Table 2:** Selected factors associated with receiving timely the birth dose of hepatitis B vaccination among children aged 0-23 months, 2014: multivariate logistic regression analysis (n=1382).

Characteristics	OR (95% CI)
Sex	
Male	1
Female	1.03(0.79,1.34)
Low birth weight	
Yes	
No	2.13*∗*(1.16,3.89)
Ethnicity	
Minority	1
Kinh/Hoa	3.15*∗∗∗*(2.04,4.88)
Area	
Rural	1
Urban	1.25(0.86,1.82)
Mother's age	
<20	1
20-35	2.24*∗∗*(1.26,3.98)
36-49	2.17*∗*(1.1,4.31)
Mother's education	
Primary or less	1
Lower secondary	1.68*∗*(1.11,2.53)
Upper secondary and tertiary	1.74*∗*(1.13,2.67)
Mother's wealth status	
1st quintile (poorest)	1
2nd quintile	1.65*∗*(1.06,2.56)
3rd quintile	1.18(0.73,1.9)
4th quintile	2.06*∗*(1.17,3.64)
5th quintile (richest)	1.17(0.65,2.11)

OR: Odd ratio; CI: confidence interval

^*∗*,*∗∗*,*∗∗∗*^: significant at 0.05, 0.01, and 0.001, respectively.

## Data Availability

The MICS datasets existing are open to public; all users were allowed to free access after requesting to use. The raw data of the 2014 survey round of MICS was obtained with the approval to use the data for this study. UNICEF MICS encourages all users to share the research findings. The details of the MICS dataset source are described in UNICEF website (http://mics.unicef.org/).
